# Stability-Indicating Spectrophotometric and TLC Densitometric Validated Methods for Simultaneous Assay of Salicylamide and Ascorbic Acid in the Presence of Salicylic Acid: Greenness Assessment and Practical Applicability

**DOI:** 10.3390/ph19070980

**Published:** 2026-06-24

**Authors:** Omkulthom Al kamaly, Saja A. Althobaiti, Maimana A. Magdy, Nourudin W. Ali, Hala E. Zaazaa, Mohamed Abdelkawy, Mohammed Gamal, Maha M. Abdelrahman

**Affiliations:** 1 Department of Pharmaceutical Sciences, College of Pharmacy, Princess Nourah bint Abdulrahman University, Riyadh 11671, Saudi Arabia; 2 Department of Chemistry, College of Science and Humanities in Al-Kharj, Prince Sattam Bin Abdulaziz University, Al-Kharj 11942, Saudi Arabia; 3 Pharmaceutical Analytical Chemistry Department, Faculty of Pharmacy, Beni-Suef University, Alshaheed Shehata Ahmad Hegazy St., Beni-Suef 62514, Egypt; 4 Pharmaceutical Analytical Chemistry Department, Faculty of Pharmacy, Cairo University, Kasr-El-Aini, Cairo 11562, Egypt

**Keywords:** salicylamide, ascorbic acid, salicylic acid, first derivative ratio technique, mean-centered ratio spectra, TLC densitometry

## Abstract

**Objectives**: Three stability-indicating analytical methods featuring outstanding sensitivity, selectivity, and precision were set up for the quantification of salicylamide (SAD) and ascorbic acid (ASC) in the presence of salicylic acid (SAL), which represents a possible impurity and degradation product of SAD. The aim was to develop sensitive, selective, precise, and eco-friendly assays appropriate for routine quality control of pharmaceuticals. **Methods**: Method (A) was a spectrophotometric technique of a successive derivative of ratio spectra built upon a two-step derivatization of ratio spectra utilizing double-distilled water as a solvent. SAD was quantified at 247.2 nm and 257.0 nm, and ASC at 251.8 and 259.8 nm, while SAL was quantified at 305.6 nm. Technique (B) relied on ratio spectra for the mean centering analytical process applied via two sequential stages, where the amplitudes derived after the second ratio spectra of the mean centering have been recorded on 291.0, 266.0, and 241.0 nm for SAD, ASC, and SAL, in that order. Method (C) involved TLC densitometric analysis, in which the separation was carried out upon plates of silica gel with chloroform–hexane–methanol–acetone–formic acid (5:3:2:1:0.2, in volumes) as a mobile phase, monitored by densitometric detection at 240 nm. The linear relationships were observed over concentration ranges of (0.2–2 µg/band) for SAD with ASC and (0.1–1 µg/band) for SAL. Validation of the presented techniques was performed in accordance with ICH strategies. **Results**: These developed techniques have been effectively analyzed for SAD with ASC in pharmaceutical dosage forms with non-interfering ingredients. A statistical comparison with the previously used HPLC technique revealed no considerable difference in terms of accuracy and precision. Greenness assessment using the AGREE platform produced scores of 0.72 for the spectrophotometric approach (benefiting from aqueous solvent) and 0.62 for HPTLC (limited by chloroform). Practical applicability (BAGI = 80 for both spectrophotometry and HPTLC) and overall quality indices (CACI = 83 for spectrophotometry; 80 for HPTLC) supported routine QC suitability. **Conclusions**: The three developed stability-indicating methods are accurate, precise, and selective for simultaneous assay of SAD and ASC in the presence of SAL and are suitable for quality control use. The spectrophotometric procedures combine high analytical performance with an improved environmental profile, while HPTLC offers comparable analytical reliability with slightly lower greenness due to organic solvent use.

## 1. Introduction

**Figure 1:**
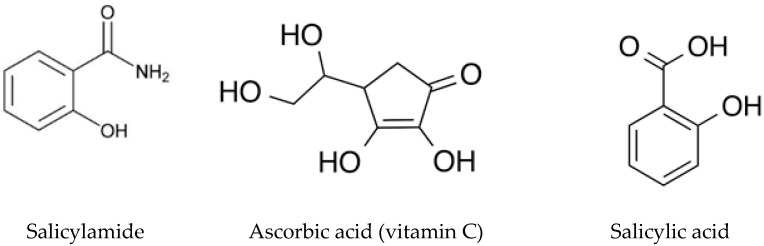
Chemical structure of salicylamide, ascorbic acid (vitamin C) and salicylic acid.

Ascorbic acid (Vitamin C), chemically identified as” 2,3-didehydro-L-threo-hexono-1,4-lactone”, is usually described as ascorbic acid (ASC) [1] (Figure 1). As a water-soluble antioxidant, it plays a vital biochemical role in collagen synthesis and in maintaining the integrity of connective tissues. From a therapeutic perspective, (ASC) is indispensable for the prevention and treatment of vitamin C insufficiency, particularly scurvy [2].

Official compendia, including both the United States Pharmacopeia (USP) [3] and British Pharmacopoeia (BP) [4], describe titrimetric assay procedures to quantify ASC at both raw materials and finished pharmaceutical formulations. Beyond these official methods, a wide range of analytical methods has been stated for determining them, such as spectrophotometry [5,6,7], spectrofluorometric [8], voltammetry [9,10,11,12], high-performance liquid chromatography (HPLC) [13,14,15,16,17,18,19,20], with capillary electrophoresis [21,22].

What is traditionally named salicylamide (SAD), also known chemically as “2-hydroxybenzamide” [1] (Figure 1), exhibits marked pain-killing, antipyretic, and anti-inflammatory effects. It is clinically employed for the relief of pain and high temperature as well as for managing provocative syndromes, including rheumatoid arthritis plus osteoarthritis [2]. The USP [3] recommends an anhydrous medium titrimetric assay to detect SAD in its initial substance form. Additionally, several investigative procedures were developed to quantify it in medicinal preparations and organic matrices. These methods include spectrophotometric [23,24,25], spectrofluorimetric [26,27], high-performance thin-layer chromatographic (HPTLC) [28,29], HPLC [30,31,32], and capillary-based electrophoretic techniques [33].

Salicylic acid has been documented as a potential degradation product and impurity of salicylamide [34], Figure 1. Pharmacologically, it functions as a mild irritant, and dermal exposure may induce local inflammatory reactions. Owing to its significant percutaneous absorption, excessive topical application can result in systemic salicylate toxicity, with severe adverse outcomes, particularly among pediatric populations, reported in the literature [2].

ASC and SAD are co-formulated in the commercial preparation Cidal C^®^ tablets, which are described for the management of common cold symptoms associated with fever and musculoskeletal discomfort. An inclusive survey of the published literature reveals that only a single HPLC technique was designated for the concurrent analysis of (SAD with ASC) in a binary mixture [35]. Notably, non-analytical methodology was described for the concurrent assessment of SAD and ASC in combination with salicylic acid as a potential impurity or degradation product of salicylamide [34]. Therefore, the development of selective, stability, and environmentally sustainable analytical strategies for their simultaneous quantification remains of considerable analytical and pharmaceutical interest. In this investigation, environmentally considerate and stability-assessing spectrophotometric methods with the TLC densitometric approaches were systemically established for the specific resolution and quantification of salicylamide (SAD) and ascorbic acid (ASC) co-existing with the process-related impurity together with its main impurity and primary breakdown product of salicylamide, namely salicylic acid. The proposed methodologies were specifically designed to overcome spectral and chromatographic interference without the necessity for preliminary separation or complex extraction procedures. Comprehensive corroboration of the established approaches has been conducted based on the regulatory recommendation established by the International Council for Harmonization (ICH) for analytical method validation [36].

## 2. Results and Discussions

Ensuring the quality of pharmaceutical products requires accurate measurement of the active drug substance as well as evaluation of its purity, stability, and related impurities. Impurities, even at low concentrations, may negatively affect drug safety and therapeutic performance. For this reason, monitoring and controlling impurities has become a fundamental requirement in pharmaceutical quality control industries. Salicylic acid is considered the main impurity of salicylamide and is also the primary degradation product. It is well known for its keratolytic efficacy [37]. To date, no published assay has reported the concurrent assay of the binary mixture of salicylamide and ascorbic acid in the presence of salicylic acid. Therefore, there is a great need for reliable analytical techniques capable of accurately determining salicylamide together with its related impurity and degradation product within pharmaceutical preparations.

The simplicity, rapidity, and affordability of spectrophotometric techniques make them useful as stability-indicating instruments. Their conventional lack of selectivity is successfully overcome when paired with contemporary data processing methods like derivative spectroscopy and chemometrics, making them a potent and long-lasting option for stability testing, particularly in quality control settings [38,39,40].

In this study, new spectrophotometric and TLC densitometric techniques were developed to be more sensitive and selective than the previously described HPLC technique. These proposed approaches allow the concurrent quantification of salicylamide and ascorbic acid in the presence of salicylic acid. In addition, they enable the quantification of salicylic acid itself, enhancing their suitability for repetitive application in quality control industries.

### 2.1. Successive Derivative Ratio Spectra Method

The proposed successive derivative proportion spectrophotometric technique was developed by studying and optimizing several factors, including the choice of solvent, type and concentration of divisor, and instrumental factors. Variable solvents (methanol, 0.1 N NaOH, water, and 0.1 N HCl) were tried to achieve better separation of the overlapping spectra of salicylamide (SAD) and salicylic acid (SAL). Water provided the clearest differentiation between these closely related spectra. Various values of Δλ and scaling factors were also tested, with optimal results achieved at Δλ = 4 and a scaling factor of 10.

**Figure 2:**
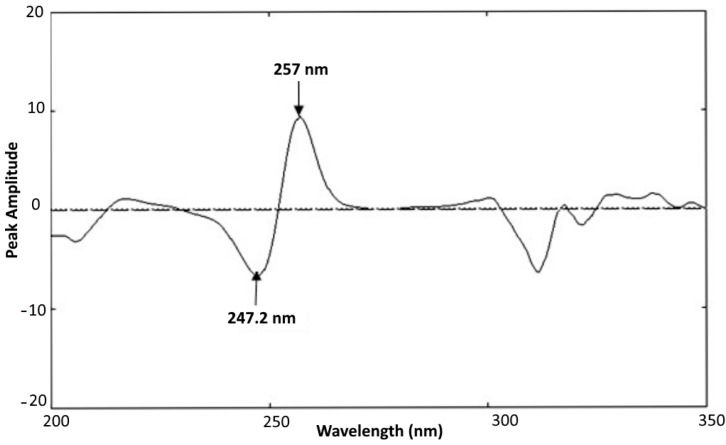
Successive derivative ratio spectra of 10 µg·mL^−1^ of salicylamide using double-distilled water as a solvent.

For the quantification of SAD, various concentrations of ascorbic acid (ASC) and salicylic acid (SAL) were tested as a divisor. In UV-VIS spectrophotometry, the divisor is a spectral key used to divide out (cancel) the known pattern of an interfering component, so that the component of interest can be seen clearly after taking derivatives. The high performance has been achieved using 20 µg/mL of ASC as a divisor. Using this setup, successive derivatives of the ratio spectra for SAD were recorded in the 2–20 µg·mL^−1^ concentration range, and the peak amplitudes were recorded at 247.2 and 257 nm; Figure 2.

**Figure 3:**
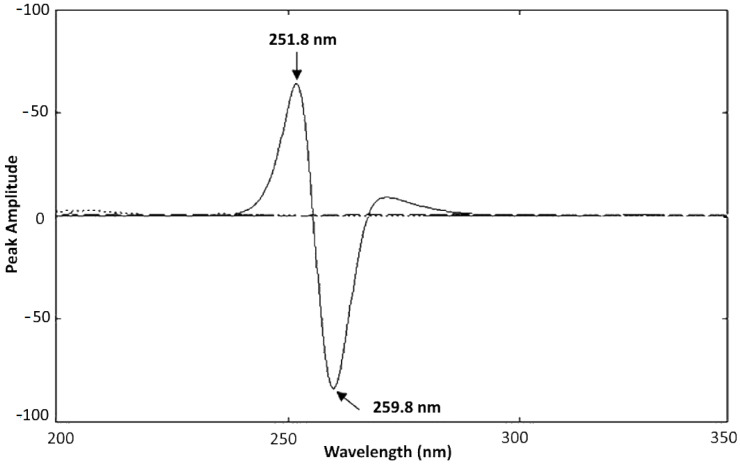
Successive derivative ratio spectra of 10 µg·mL^−1^ of ascorbic acid using double-distilled water as a solvent.

Similarly, for ASC quantification, various concentrations of SAD and SAL were tested as a divisor. The optimum end results were achieved with 20 µg/mL of (SAD) as a divisor. Successive derivative percentage spectra for ASC were then achieved in the 2–20 µg·mL^−1^ range, with peak amplitudes at 251.8 and 259.8 nm, illustrated in Figure 3.

**Figure 4:**
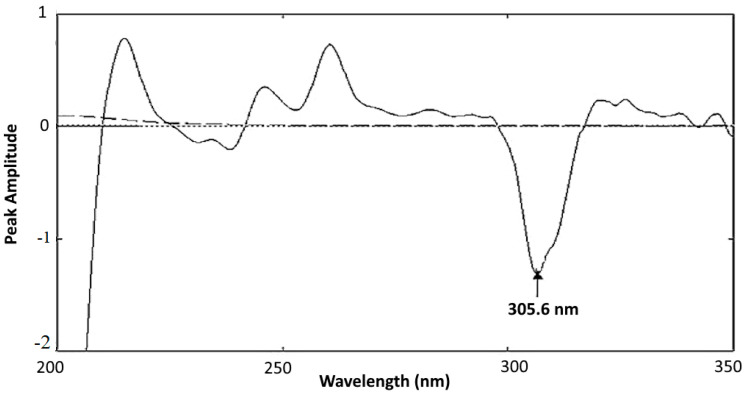
Successive derivative ratio spectra of 10 µg·mL^−1^ of salicylic acid using double-distilled water as a solvent.

For SAL analysis, various concentrations of ASC and SAD have been examined as a divisor. The maximum end results were achieved after using 20 µg/mL of (ASC) as a divisor. Successive derivative ratio spectra of SAL were achieved in the 2–20 µg·mL^−1^ concentration range, and the peak amplitude was measured at 305.6 nm Figure 4. Linear relationships were observed among peak amplitudes and concentrations for all three compounds. The regression formulas were estimated as follows:

- SAD: P. A = 0.6346CSAD − 0.0808  r = 0.9998,    at 247.2 nm
- SAD: P. A = 0.4977CSAD + 0.2217  r = 0.9998,    at 257.0 nm
- ASC: P. A = 5.5406CASC + 8.8707  r = 0.9999,    at 251.8 nm
- ASC: P. A = 7.2287CASC + 11.3708  r = 0.9999,  at 259.8 nm
- SAL: P. A = 0.1027 CSAL − 0.0069  r = 0.9998,    at 305.6 nm

P.A. represents the peak amplitude at the designated wavelengths. C-SAD, C-ASC and C-SAL represent the concentrations of (SAD), (ASC) and (SAL) µg·mL^−1^, where r denotes the correlation coefficient value.

### 2.2. Mean Centering of Ratio Spectra Spectrophotometric Method (MCR)

The mean centering of ratio spectra (MCR) technique was employed in this study as a selective spectrophotometric approach. The theoretical background and mathematical basis of this method were previously described by Afkhami and Bahram [41]. Owing to its capability to analyze complex systems, it has been successfully utilized for the resolution of binary and ternary mixtures, even in samples containing unknown matrices [41]. To ensure optimal performance of the MCR method, the influence of divisor concentration on method selectivity was carefully examined. Several concentrations (4, 6, 8, 14, and 20 µg·mL^−1^) of SAD, ASC and SAL were investigated using their normalized spectra. The obtained results demonstrated that divisor concentration plays a critical role in achieving reliable selectivity. The most reproducible and accurate responses were achieved when 20 µg/mL was used as the divisor concentration. Specifically, 20 µg·mL^−1^ of ASC and SAL were selected for SAD determination, 20 µg·mL^−1^ of SAD and SAL were used for ASC assay, and 20 µg·mL^−1^ of SAD and ASC were applied for SAL analysis.

**Figure 5:**
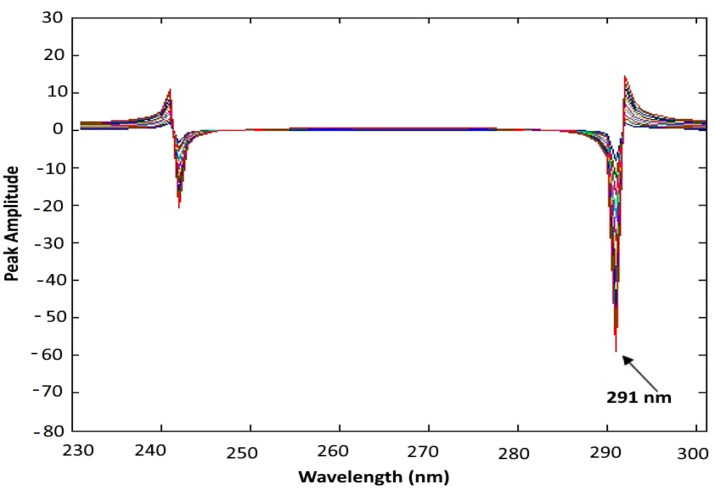
The mean-centered first ratio absorption spectra of salicylamide in the range of 2–20 µg·mL^−1^ using double-distilled water as a solvent.

**Figure 6:**
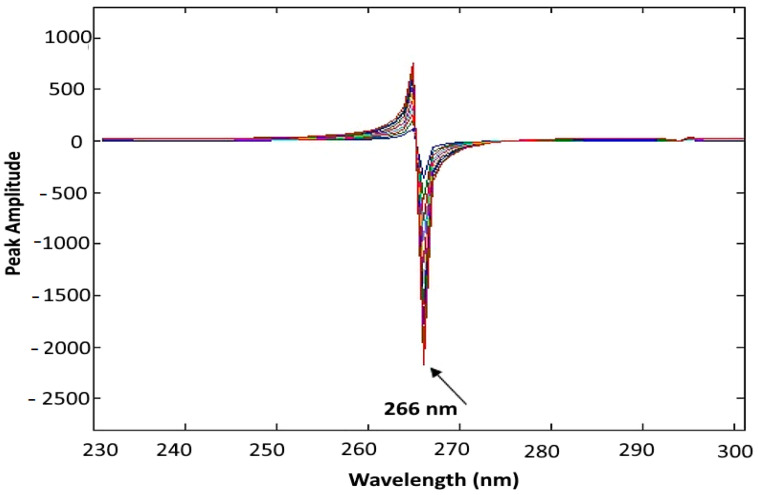
The mean centered first ratio absorption spectra of ascorbic acid in the range of 2–20 µg/mL using double-distilled water as a solvent.

**Figure 7:**
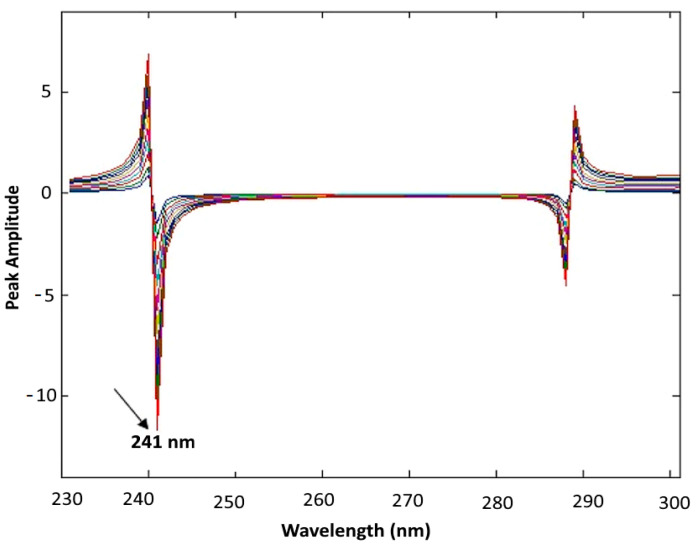
The mean-centered first ratio absorption spectra of salicylic acid in the range of 2–20 µg/mL using double-distilled water as a solvent.

The linearity of the developed MCR method was assessed for each component, and satisfactory linear relationships were obtained within the concentration range of 2–20 µg·mL^−1^. Measurements were performed at 291 nm for SAD, 266 nm for ASC, and 241 nm for SAL (Figure 5, Figure 6 and Figure 7). The corresponding regression formulas were derived as follows:

- SAD: P. A = 2.9008 CSAD + 0.5566    r = 0.9998,  at 291 nm
- ASC: P. A = 101.0653 CASC + 159.4013  r = 0.9999,  at 266 nm
- SAL: P. A = 0.5803 CSAL − 0.0632    r = 0.9998,  at 241 nm

P.A. denotes the measured peak amplitude at the nominated wavelengths. C-SAD, C-ASC and C-SAL represent the corresponding concentrations in µg·mL^−1^, and r represents the correlation coefficient.

### 2.3. TLC Densitometric Method

In the present analytical assay, TLC densitometry was adopted as a chromatographic tool for both resolution and quantitative analysis of the investigated ternary mixture. The separation step was first achieved on TLC plates using an optimized developing system, after which the resolved bands were scanned using densitometry. The recorded optical responses were used for quantification through calibration graphs constructed from standard solutions chromatographed under identical experimental conditions [42,43].

To maximize the studied components’ separation, it was essential to examine the various parameters’ efficacy. Reviewing the maximum separation with optimum parameters was approved as follows:

A. Optimization of the mobile phase

**Figure 8:**
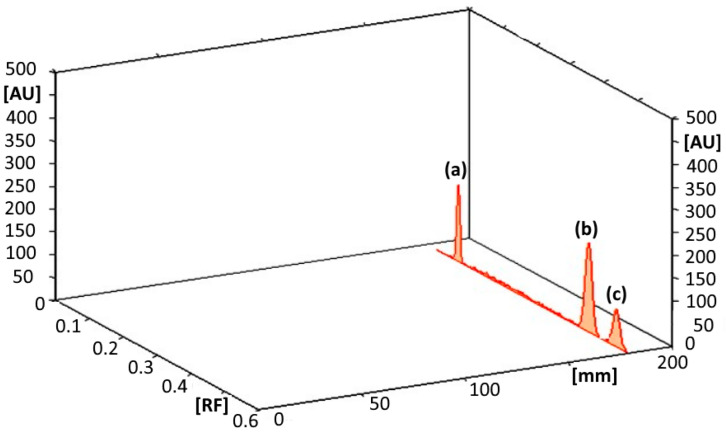
Thin layer chromatogram of separated peaks of (a) ascorbic acid, (b) salicylamide and (c) salicylic acid using hexane/chloroform/acetone/methanol/formic acid (5:3:2:1:0.2, by volume) as a developing liquid system.

Several solvent systems with altered conformations and ratios have been examined to obtain satisfactory separation of the three analytes. The most suitable mobile phase consisted of hexane/chloroform/acetone/methanol/formic acid (5:3:2:1:0.2, in volumes). This selected solvent provided convenient separation within the ternary mixtures with acceptable R_f_ values with no observed tailing of the resolved bands (Figure 8).

B. Optimization of Band Parameters

The effect of bandwidth and interband spacing was investigated to achieve symmetrical and sharp peaks. A bandwidth of 6 mm combined with 5 mm spacing between adjacent bands was found to produce optimal peak shape and resolution.

C. Selection of Scanning Wavelength

Various detection wavelengths (230, 240, and 254 nm) were evaluated. Among them, 240 nm gave the highest sensitivity for all analytes, with sharp and symmetrical peaks and minimal baseline noise. Therefore, this wavelength was selected for subsequent measurements.

D. Adjustment of slit dimensions

Proper slit dimensions are essential to ensure complete coverage of the chromatographic band during scanning without interference from neighboring bands. After evaluating several options, a slit size of 6 mm × 0.3 mm was chosen, as it provided maximum sensitivity and reliable detection.

#### 2.3.1. System Suitability Assessment

In accordance with USP guidelines [3], system suitability factors were evaluated to confirm the adequacy of the chromatographic system. Resolution (Rs), selectivity factor (α), and peak symmetry have been calculated. The obtained results showed resolution values exceeding 2, selectivity factors greater than 1, and acceptable symmetry factors, confirming proper system performance (Table 1).

#### 2.3.2. Calibration and Linearity Assessments

A direct proportional relationship was obtained between concentration and the integrated peak area (×10^−4^). The linear response covered the concentration ranges of 0.2–2 µg/band to ASC, 0.22 µg/band to SAD, and 0.1–1 µg/band to SAL, based on calibration plots constructed from the recorded peak areas.

The three regression formulas were expressed as follows:

- SAD: P.A. = 3.4966CSAD + 1.7965 (r = 0.9996) at 240 nanometers
- ASC: P.A. = 1.3324 CASC − 0.1348 (r = 0.9997) at 240 nanometers
- ASL: P.A. = 2.5354 CSAL − 0.0381 (r = 0.9996) at 240 nanometers

PA represents the integrated peak area (×10^−4^); CSAD, CASC, and CSAL denote the concentration in µg/band; and r denotes the correlation coefficient value.

Application of the developed chromatographic technique demonstrated its suitability for analyzing laboratory-prepared mixtures. Additionally, the previously described spectrophotometric techniques showed sufficient selectivity to quantify SAL at 10% of SAD concentration (Table 2).

These techniques were successfully suitable for the detection of SAD with ASC in Cidal C^®^ tablets. Recovery studies using the standard addition technique confirmed the lack of interference from formulation excipients (Table 3). The active ingredients (ascorbic acid and salicylamide) were effectively determined in their pharmaceutical product (Cidal C^®^), as described in Table 3. Although salicylic acid was not detected as a degradation product in the current formulation, the primary aim of this analytical work was to develop a procedure capable of detecting it in both the raw material and the final dosage form, particularly in cases of poor storage conditions or product expiry.

Validation of the novel techniques was performed with respect to ICH guidelines [36]. The obtained data for accuracy, repeatability, and intermediate precision are summarized in Table 4. Additionally, the results of RSD% values less than 1.1 in Supplementary Table S1 confirm the reliability of the TLC method for the simultaneous assay of the three compounds. Furthermore, statistical comparisons with the described HPLC technique [35] using a t-test of the student and F-test indicated no significant difference between the investigated techniques in terms of precision and accuracy, as the calculated values were lower than the theoretical limits (Table 5 and Table 6). The chromatogram for the reported HPLC technique [35] was displayed in Supplementary Figure S2.

### 2.4. Methods’ Greenness Assessment and Practical Applicability Evaluation

#### 2.4.1. AGREE Tool

Based on the twelve codes of Green Analytical Chemistry, AGREE (Analytical GREEnness) is a comprehensive metric that assesses an analytical method’s environmental sustainability [45]. It produces a final score between 0 and 1, represented as a circular clock-like pictogram, making it possible to estimate the overall greenness of a method quickly and easily [45,46].

The novel spectrophotometric method attained an AGREE score of 0.72, while the developed TLC method acquired a score of 0.62. The higher score achieved by the spectrophotometric method is attributed to the use of pure water as an environmentally friendly solvent. In contrast, the lower score obtained for the TLC method results from the utilization of chloroform, a non-green solvent (Table 7).

#### 2.4.2. BAGI Tool

An analytical method’s practical applicability is valued using a statistic called the Blue Applicability Grade Index (BAGI). Important characteristics, including analytical time, sample throughput, instrumentation needs, and operational simplicity, are evaluated. Analysts can rapidly ascertain whether a procedure is feasible for ordinary laboratory use by viewing the results as a blue pictogram with a final score (25.0–100.0) [47].

Despite having differing operating strengths, the spectrophotometric and HPTLC procedures share a similar degree of practical applicability for normal use, as indicated by their respective BAGI scores of 80. While HPTLC provides increased sample throughput through simultaneous analysis, the spectrophotometric approach shines in operational simplicity and speed. This score demonstrates that both approaches balance performance and practicality through their distinct procedural advantages, making them suitable for quality control environments (Table 7).

#### 2.4.3. CACI Evaluation

A scoring system that evaluates an analytical method’s overall quality quantitatively is the Click Analytical Chemistry Index (CACI). It assesses important performance pillars, such as environmental safety, cost-effectiveness, simplicity, and validation parameters, and combines them into a single objective score (usually between 0 and 100). This makes it simple for analysts to compare approaches and choose the one that best strikes a balance between sustainability, practicality, and analytical rigor [48].

The spectrophotometric method’s CACI score of 83 denotes a very dependable and solid analytical process. Its great linearity, high precision, and sufficient sensitivity for routine quantitative analysis are confirmed by this excellent assessment, which positions it as an effective and economical instrument for quality control laboratories with low interference susceptibility. The HPTLC method’s CACI score of 80 indicates a reliable and appropriate analytical technique with clear benefits. The method’s value for simultaneous analysis and impurity profiling with high sample throughput is highlighted by the somewhat lower score, which reflects the slightly higher variability inherent to this multi-step methodology, yet confirms appropriate accuracy, specificity, and precision (Table 7).

## 3. Materials and Methods

### 3.1. Instruments

Analytical assessment has been performed via a double-beam UV–visible spectrophotometer (Shimadzu, Kyoto, Japan; model UV-1601 PC) equipped with 1 cm quartz cuvettes, plus interfaced with an IBM-compatible processor. Spectral data were recorded and processed using UVPC spectroscopy software (version 3.7). Multivariate evaluation of the data has been carried out with the aid of PLS-Toolbox 2.0 operating beneath “MATLAB version 6.5” [49]. The instrument was adjusted to a spectral slit width of 2 nm, while wavelength scanning was conducted at a rate of 2800 nm per minute.

UV lamp emitting at 254 nanometer (Jelight Company Inc., Irvine, CA, USA) served as radiation source. Thin-layer chromatography (TLC) measurements were achieved by usage of Camag TLC Scanner 3 densitometer (Muttenz, Switzerland). The operational parameters for scanning and sample application were optimized as follows:

Slit dimensions of (6 mm × 0.3 mm)

With scan velocity of (20 mm/s)

Spraying at a rate of (10 s/µL)

Statistics were collected with a resolution of 100 µm/step.

To ensure reproducible peak integration and reliable quantitation.

- Aluminum-backed TLC analyses utilized plates layered with silica gel (60F254) (20 × 20 cm), (Fluka, Sigma-Aldrich Chemie GmbH, Steinheim, Germany).
- Sample application was accomplished using TLC linomat IV semi-automatic applicator, Camag (Muttenz, Switzerland), equipped with a calibrated 100 µL microsyringe to guarantee precision.

### 3.2. Materials

#### 3.2.1. Standard and Pure Compounds

Salicylamide and ascorbic acid reference powders have been obtained from Chemical Industries Development (CID) Company, Cairo, Egypt. The certified assay results of purity were confirmed by a previously reported HPLC technique [35], which were 100.46 ± 1.288 and 99.41 ± 1.475, respectively. Salicylic acid was acquired from Sigma-Aldrich Company (Steinheim, Germany), indicating a purity of 99%.

#### 3.2.2. Pharmaceutical Product

A commercially available tablet form, Cidal C^®^ (Batch No. 121135W) containing 500 mg of SAD and 50 mg of ASC per tablet and non-active excipients (including microcrystalline cellulose + magnesium stearate + stearic acid + silicon dioxide + croscarmellose sodium) was purchased and analyzed. The product was obtained from the “Chemical Industries Development (CID) Company, Cairo, Egypt.

#### 3.2.3. Reagents and Solvents

All solvents and reagents had an analytical evaluation grade and were utilized directly as received with no extra purification steps. Hexane, acetone, chloroform, formic acid, and also methanol were bought from El-Nasr Pharmaceutical Chemicals Company, Heliopolis, Cairo, Egypt. Moreover, double-distilled (H_2_O) was supplied by Otsoka Pharmaceuticals, 6th of October City, Giza, Egypt.

### 3.3. Standard Solutions

#### 3.3.1. Concentrated Stock Solutions

Primary solutions of salicylamide (SAD), ascorbic acid (ASC), and salicylic acid (SAL) were individually dissolved in methanol to give a final concentration of 1 mg/mL. Accurately weighed portions (0.1 g) of each compound were placed into separate 100 mL calibrated flasks, dissolved in a suitable volume of methanol with agitation, then made up to the mark with a similar solvent.

#### 3.3.2. Diluted Working Solutions

For analytical applications, secondary solutions (100 µg·mL^−1^) have been freshly diluted from the corresponding concentrated stock solutions. Aliquots (10 mL) were quantitatively transferred to standard volumetric flasks and completed to the marked volume using double-distilled water purified by distillation for spectrophotometric measurements, while methanol was employed for TLC densitometric analysis.

### 3.4. Laboratory-Prepared Mixtures

A set of mixtures prepared in the laboratory, having several proportions of SAD, ASC, and SAL, was prepared. For analysis using spectrophotometry, the mixtures were obtained by appropriate dilution of their working solutions using double-distilled water as the solvent. In contrast, mixtures intended for TLC densitometric analysis were prepared utilizing methanol as a stock standard solution and diluting medium.

## 4. Descriptions of Analytical Laboratory Procedures

### 4.1. Spectral Features of Salicylamide, Ascorbic Acid, and Salicylic Acid

Solutions of SAD, ASC, and SAL (10 µg·mL^−1^) were individually scanned using a UV–Visible spectrophotometer. Their absorption spectra were collected across the wavelength region (200 to 400 nanometers), employing double-distilled H_2_O as a blank. The obtained absorption profiles are presented in Supplementary Figure S1.

### 4.2. Successive Derivative Ratio Spectrophotometric Technique

Separate portions corresponding to 20–200 µg of each drug were transferred into volumetric flasks of 10 mL capacity, followed by dilution to the marked volume with double-distilled water. After that, the zero-order absorption spectra were subsequently measured within the 200 to 400 nanometer wavelength range.

After SAD quantification, the obtained spectra have been divided by the ASC standard spectrum (20 µg·mL^−1^) to generate the primary ratio spectra. These spectra had been subsequently converted into their first derivative form. The resulting derivative spectra were further divided by (d/dλ)(SAL/ASC) derived from the first derivative ratio spectra of 20 µg/mL of both SAL and ASC, producing the second ratio spectra. Finally, the first derivative of these spectra was calculated using Δλ = 4.

For ASC determination, the spectra were divided by the spectrum of SAD (20 µg/mL), followed by derivatization. The obtained derivative spectra were subsequently distributed by (d/dλ)(SAL/SAD) for the generation of the second ratio spectra.

As for SAL, the spectra were subjected to division using the spectrum of ASC (20 µg/mL). The obtained derivative vectors were subsequently subjected to division by (d/dλ) (SAD/ASC) obtained from equal concentrations of both compounds. In addition, calibration graphs were assembled by involving the measured amplitudes at 247.2–257 nm (SAD), 251.8–259.8 nm (ASC), and 305.6 nanometers (SAL) plotted as a function of the drug levels.

### 4.3. Mean Centering of Ratio Spectra Method (MCR)

Separate aliquots of 20–200 µg from the working solutions of SAD, ASC, and SAL (100 µg/mL) were put into 10 mL volumetric flasks, and double-distilled water was used to dilute the solutions to the appropriate level. The range of their absorption spectra was 200–400 nm.

To get the initial ratio spectra for SAD analysis, the spectra were subjected to division by the ASC spectrum (20 µg/mL). Mean-centering was then applied to the spectra. Dividing the produced vectors by the mean-centered ratio, the latter one was obtained. The second ratio spectra were calculated by dividing the produced vectors by the mean-centered ratio of αSAL/αASC, and then another mean-centering step was performed. The spectra were first split by the SAD spectrum (20 µg/mL) for ASC. The mean-centered ratio spectra that resulted were divided by the mean-centered ratio αSAL/αSAD, producing the latter one’s mean-centered ratio spectra. The spectra were split by the ASC spectrum (20 µg/mL) for SAL. The resultant vectors were divided by the mean-centered ratio αSAD/αASC and then mean-centered once more after the ratio spectra had been mean-centered.

The mean-centered second ratio spectra’s amplitudes were determined at: (291 nanometer) for SAD, (266 nanometer) for ASC, Plus (241 nanometer) for SAL, and calibration plots were established by plotting these amplitudes versus the respective concentrations.

### 4.4. TLC Densitometric Method

SAD, ASC, and SAL working solutions were divided into 10 mL flasks and diluted with methyl alcohol. Using a Camag Linomat IV applicator, 10 µL of each prepared solution was placed in bands of 6 mm width on TLC plates (20 × 10 cm, 250 µm thick). The bands were placed 10 mm from the lower edge and plate sides, with a 5 mm gap between each band. Using the ascending approach, chromatographic development was carried out up to 8 cm inside a developing chamber that had been saturated with the mobile phase vapors for thirty minutes. The mobile phase was made up of the following: chloroform/hexane/acetone/methanol/formic acid (3:5:2:1:0.2, in volumes).

At 240 nm, densitometric scanning was performed, and the appropriate peak regions were noted. Plotting integral peak area (×10^−1^) versus drug concentrations and computing the corresponding regression formulas yielded calibration curves.

### 4.5. Assay of Laboratory-Prepared Mixtures of SAD, ASC and SAL

From their working solutions, synthetic combinations with different concentrations of SAD, ASC, and SAL were created. The diluting solvent for spectrophotometric methods was double-distilled water, whereas methanol was employed for the TLC densitometric analysis. The prepared mixtures were analyzed following the previous described procedures illustrated in Section 3.2, Section 3.3 and Section 3.4.

### 4.6. Application to Pharmaceutical Formulation (Cidal C^®^ Tablets)

Ten Cidal C^®^ tablets (each tablet weighed approximately 1 gm) were pulverized to a fine powder and thoroughly blended to achieve homogeneity. A precisely weighed portion of the powdered tablets (200 mg powdered tablets), containing “500:50 mg” of SAD: ASC, respectively, was transferred into a 100 mL calibrated flask. Approximately 75 mL of methanol was added, and the final solution was treated with ultrasonic waves for 30 min to confirm maximal extraction of the active constituents. The resulting mixture was then filtered, and the filtrate volume was completed to the final mark with methyl alcohol.

This solution corresponded to a working concentration of 100 µg/mL for ASC (and 1000 µg/mL of SAD), whereas an appropriate dilution step was performed to get a 100 µg/mL working solution of SAD (by diluting 10 mL from the stock solution into 100 mL). For spectrophotometric measurements, double-distilled water was selected as the solvent, while methanol served as the solvent in TLC analysis. Quantitative determination of both compounds was achieved according to the aforementioned procedures mentioned in divisions 4.2, 4.3, and 4.4, and their concentrations were computed from the relevant regression formulas.

## 5. Conclusive Remarks

The industrialized analytical techniques provide reliable, highly responsive, and specific approaches for the concurrent quantification of salicylamide plus ascorbic acid together with salicylic acid. Their successful application to both bulk powder and pharmaceutical formulations confirms their suitability for routine pharmaceutical analysis. Spectrophotometric techniques offer notable advantages due to their simplicity, ease of operation, and rapid performance. Greenness (AGREE scores over 0.6) and practical applicability (BAGI/CACI scores ≥ 80) evaluations affirm both methods as sustainable, feasible, and superior choices for routine stability-indicating assays in pharmaceutical quality control. For routine quality control of pharmaceutical formulations, these features make them appealing substitutes for more complex chromatographic analytical procedures.

Furthermore, when compared to the previous HPLC technique, the TLC densitometric method offers significant practical advantages. It makes it possible to evaluate multiple samples at once with less mobile phase, which speeds up analysis and lowers operating costs while preserving sufficient sensitivity and selectivity.

In terms of sensitivity, the TLC densitometric method proved to be the most sensitive among the evaluated techniques, outperforming both the successive derivative ratio spectra method and the mean centering of ratio spectra spectrophotometric method. Furthermore, the TLC procedure offers a practical advantage in throughput, as it permits the simultaneous assessment of up to 20 samples per run, thereby considerably reducing total analysis time.

Regarding instrumental accessibility, however, TLC densitometers are not universally available in conventional analytical laboratories. In contrast, UV-Vis spectrophotometers—the instruments employed for both spectrophotometric methods—are more widely accessible and relatively straightforward to operate. It should also be noted that the mean centering approach requires specialized software (e.g., MATLAB), whereas the successive derivative ratio spectra method does not necessitate such additional computational software.

## Figures and Tables

**Table 1:** Evaluation parameters of system suitability for the TLC densitometric technique applied to quantification of salicylamide, ascorbic acid with salicylic acid.

Evaluation Parameters	Calculated Value					Reference and Literature Value [44]
	ASC	SAD			SAL	
R_f_	0.15	0.5			0.55	
Resolution (Rs)	14.86		2.75			R > 1.5
Selectivity coefficient factor (α)	3.33		1.1			>1
Peak Symmetry factor	0.99	1.02		1.05		≈1

**Table 2:** Quantitative assay of salicylamide, ascorbic acid and salicylic acid in laboratory combinations by the recommended successive derived proportion and mean-centered ratio spectra spectrophotometric techniques.

SAL%	Analyte Amount µg·mL^−1^			Recovery *%							
				Successive Derived Ratio Spectra Spectrophotometric Technique					Ratio Spectra Analysis Using Mean Centering Spectrophotometry		
	SAD	ASC	SAL	SAD		ASC		SAL	SAD at 291 nm	ASC at 266 nm	SAL at 241 nm
				at 247.2 nm	at 257 nm	at 251.8 nm	at 259.8 nm	at 305.6 nm			
10	18	2	2	99.88	99.61	97.50	98.00	102.50	99.67	98.00	101.00
15	17	5	3	100.37	101.25	101.50	100.00	98.00	98.25	100.75	99.00
20	14	4	4	101.12	101.53	99.60	100.20	98.66	99.29	100.20	98.67
33	12	4	6	98.33	101.08	100.75	100.50	99.66	101.75	100.00	102.00
40	3	10	2	101.40	97.20	99.50	100.30	100.80	102.00	101.00	100.20
50	5	10	5	99.33	102.66	102.00	99.90	103.00	102.67	101.90	101.50
66	3	6	6	101.00	100.33	100.00	100.66	102.00	102.33	99.67	101.17
Mean ± SD				100.37 ± 1.104	101.08 ± 1.748	100.00 ± 1.493	100.20 ± 0.894	100.80 ± 1.947	101.75 ± 1.743	100.20 ± 1.224	101.00 ± 1.267

* All recovery values represent the average of three trials, ensuring reliability of the reported data.

**Table 3:** Application of the three analytical procedures to a commercial formulation using the standard addition approach.

Analyzed Dosage Form	Analytical Technique Applied	Analytes: Measurement Wavelength/Condition		Sample Amount Used	Recovery Obtained * % ± SD	Standard Addition Recovery ** (Mean ± SD)
Cidal C^®^ tablets (labeled to contain 500 mg SAL and 50 mg ASC per tablet: Batch No 121135W)	Sequential derivative ratio spectrophotometric technique	SAD	Measured at 247.2 nanometer	8 µg·mL^−1^	101.50 ± 1.211	100.02 ± 1.552
			Recorded 257 nanometer	8 µg·mL^1^	103.12 ± 1.031	100.97 ± 1.806
		ASC	Determined at 251.8 nanometer	2 µg·mL^−1^	104.00 ± 1.165	100.05 ± 0.565
			Measured at 259.8 nanometer	2 µg·mL^−1^	105.50 ± 1.366	100.15 ± 0.754
	Ratio-spectra mean-centering spectrophotometric approach	SAD	Monitored at 291 nanometer	8 µg·mL^−1^	102.50 ± 1.134	99.63 ± 1.444
		ASC	Recorded at 266 nanometer	2 µg·mL^−1^	105.00 ± 1.505	100.92 ± 1.290
	TLC densitometric method	SAD	Detection at 240 nanometer	0.6 µg per band	103.67 ± 1.487	100.17 ± 1.604
		ASC	Detection at 240 nanometer	0.6 µg per band	105.33 ± 1.765	100.99 ± 1.734

* Results correspond to the mean of six measurements. ** Values represent the mean of three replicate determinations.

**Table 4:** Validation data of the developed analytical techniques for quantifying salicylamide, ascorbic acid and salicylic acid.

Analytical Characteristics	Sequential Derivative Spectrophotometric Technique					Ratio Spectra Mean-Centering Spectrophotometric Approach			Thin Layer Chromatography Densitometric Analysis		
	SAD		ASC		SAL	SAD	ASC	SAL	SAD	ASC	SAL
	At (247.2) Nanometer	At (257) Nanometer	At (251.8) Nanometer	At (259.8) Nanometer	At (305.6) Nanometer						
Range	2–20 µg·mL^−1^					2–20 µg·mL^−1^			0.2–2 (µg/band)	0.2–2 (µg/band)	0.1–1 (µg/band)
Calibration Slope	0.6346	0.9477	5.5406	7.2287	0.1027	2.9008	101.0653	0.5803	3.4966	1.3324	2.5354
*Y*-axis Intercept	0.2217	−0.0808	8.8707	11.3708	−0.0069	0.5566	159.4013	−0.0632	1.7965	−0.1348	−0.03811
Regression Correlation(r)	0.9998	0.9998	0.9999	0.9999	0.9998	0.9998	0.9999	0.9998	0.9996	0.9997	0.9996
Recovery percentage (mean ± SD)	100.22 ± 0.938	99.78 ± 1.208	100.08 ± 1.003	100.05 ± 0.787	99.97 ± 1.391	100.0 ± 1.458	100.14 ± 0.947	99.86 ± 1.309	99.86 ± 1.190	100.22 ± 1.609	100.11 ± 1.417
Selectivity assessment (mean ± SD)	100.37 ± 1.104	101.08 ± 1.748	100.00 ± 1.493	100.20 ± 0.894	100.80 ± 1.947	101.75 ± 1.743	100.20 ± 1.224	101.00 ± 1.267	--	--	--
Precision expressed as (%RSD) Intra-day precision * Inter-day precision *	1.31 1.66	1.32 1.40	1.19 1.24	1.25 1.48	1.17 1.66	1.45 1.85	1.74 1.95	1.29 1.43	1.32 1.88	1.24 1.55	1.55 1.67
LOD **	0.54 (µg·mL^−1^)	0.51 (µg·mL^−1^)	0.64 (µg·mL^−1^)	0.55 (µg·mL^−1^)	0.61 (µg·mL^−1^)	0.63 (µg·mL^−1^)	0.52 (µg·mL^−1^)	0.59 (µg·mL^−1^)	0.06 (µg/band)	0.05 (µg/band)	0.04 (µg/band)
LOQ **	1.73 (µg·mL^−1^)	1.52 (µg·mL^−1^)	1.91 (µg·mL^−1^)	1.66 (µg·mL^−1^)	1.83 (µg·mL^−1^)	1.92 (µg·mL^−1^)	1.65 (µg·mL^−1^)	1.76 (µg·mL^−1^)	0.18 (µg/band)	0.21 (µg/band)	0.13 (µg/band)

* Within-day precision (*n* = 3), calculated from three replicate analyses at three concentration levels performed during a single day. Between-days precision (*n* = 3), evaluation performed across three consecutive days. ** Detection limit calculated as LOD = 3.3 × (SD of the response/slope); Quantification limit estimated as (SD of the response/slope) × 10.

**Table 5:** Comparison of statistical evaluation results achieved using the developed analytical procedures and the previously published method for the quantification of salicylamide and ascorbic acid in their pure powder form.

Items	Successive Derivative Ratio Spectra Spectrophotometric Method				Ratio Spectra Mean-Centering Spectrophotometric Approach		Thin Layer Chromatographic–Densitometric Analysis		Previously Published Analytical Technique [35] *	
	SAD		ASC		SAD at 291 nm	ASC at 266 nm	SAD	ASC	SAD	ASC
	Measured at (247) Nanometer	Measured at (257) Nanometer	Measured at (251.8) Nanometer	Measured at (259.8) Nanometer						
Mean	100.22	99.78	100.08	100.05	100.00	100.14	99.86	100.22	100.46	99.41
SD	0.938	1.208	1.003	0.787	1.458	0.947	1.190	1.609	1.288	1.475
%RSD	0.936	1.211	1.002	0.786	1.458	0.946	1.192	1.605	1.282	1.484
*n*	10	10	10	10	10	10	8	8	6	6
Variance	0.879	1.459	1.006	0.619	2.205	0.897	1.416	2.589	1.658	2.176
Calculate Student’s t statistic	0.615 (2.145) **	0.199 (2.145) **	0.164 (2.145) **	0.151 (2.145) **	0.206 (2.145) **	0.142 (2.145) **	0.633 (2.179) **	0.678 (2.179) **	-	-
Calculated –F-statistic	1.885 (3.482) **	1.137 (3.482) **	2.159 (3.482) **	3.091 (3.482) **	1.282 (3.482) **	2.423 (3.482) **	1.172 (3.971) **	1.189 (4.875) **	-	-

* Reference HPLC procedure performed using a C8 column with a mobile system composed of 0.03 M aqueous phosphate buffer: methyl alcohol mixture (45:55, in volume) with ultraviolet detection at 255 nanometer. The speed of flow was 1 mL.min^−1^. ** Values shown in parentheses correspond to the tabulated critical values of Student (t and F) tests at a significance level of *p* = 0.05.

**Table 6:** Statistical assessment of the novel analytical approaches in comparison with the reference HPLC platform for the assay of (Cidal C^®^ tablets).

Statistical Parameters	Derivative Ratio Spectrophotometric Approach				Mean Centered Ratio Spectra Spectrophotometric Technique		Densitometric TLC Procedure		HPLC-Reference Procedure [35] *	
	SAD		ASC		SAD at 291 Nanometer	ASC at 266 Nanometer	SAD	ASC	SAD	ASC
	at 247.2 Nanometer	at 257 Nanometer	at 251.8 Nanometer	at 259.8 Nanometer						
Average recovery (%)	101.50	103.12	104.00	105.50	102.5	105.00	103.67	105.33	102.50	104.50
Standard deviation (SD)	1.211	1.031	1.165	1.366	1.134	1.505	1.487	1.765	1.472	1.366
Relative standard deviation (%RSD)	1.193	0.999	1.120	1.295	1.106	1.433	1.434	1.676	1.436	1.307
*n*	6	6	6	6	6	6	6	6	6	6
Calculated Variance	1.466	1.063	1.357	1.866	1.286	2.265	2.211	3.115	2.167	1.866
Calculated *t*-value	0.642 (2.228)	1.622 (2.228)	0.229 (2.228)	1.255 (2.228)	0.404 (2.228)	0.402 (2.228)	1.489 (2.228)	0.655 (2.228)	-	-
Variance ratio (F)	1.477 (5.050) **	2.039 (5.050) **	1.373 (5.050) **	1.000 (5.050) **	1.683 (5.050) **	1.214 (5.050) **	1.021 (5.050) **	1.669 (5.050) **	-	-

* The reference procedure was performed using HPLC with a C8 stationary material and a mobile system composed of 0.03 M aqueous phosphate buffer: methyl alcohol mixture (45:55, in volume), monitored at 255 nm. The speed of flow was 1 mL.min^−1^. ** Values shown in parentheses correspond to the theoretical critical values of F and t at a 95% confidence level (*p* = 0.05).

**Table 7:** Evaluation of the novel techniques using AGREE, BAGI, and CACI tools.

Assessment Tool	Successive Derivative of Ratio Spectra and Mean Centering Methods	TLC Method
AGREE	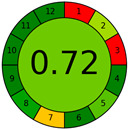	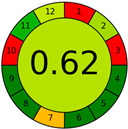
BAGI	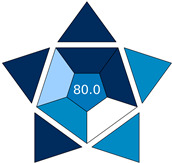	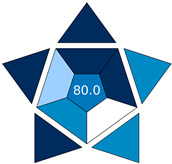
CACI	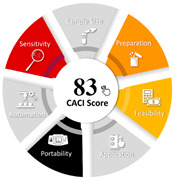	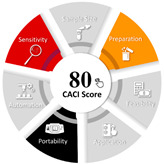

## Data Availability

The original contributions presented in this study are included in the article/Supplementary Materials. Further inquiries can be directed to the corresponding authors.

## Supplementary Materials

The following supporting information can be downloaded at: https://www.mdpi.com/article/10.3390/ph19070980/s1. Supplementary Figure S1: Zero-order absorption spectra of 10 µg·mL^−1^ of each of SAD (_), ASC (----), and SAL (….) using water solvent. Supplementary Figure S2: typical chromatogram for the lab-prepared mixture of ascorbic acid (A), salicylamide (B), and salicylic acid (C) using C8 and chromatographic conditions reported by El-Din et al. [35]. Supplementary Table S1: Results of robustness for the assay of SAD, ASC, and SAL by the new TLC densitometric technique.

## Abbreviations

The following abbreviation list is used in this paper.

ASC	Ascorbic acid
SAD	Salicylamide
SAL	Salicylic acid
QC	Quality control
TLC	Thin-layer chromatography
HPLC	High-Performance Liquid Chromatography
HPTLC	High-Performance Thin-Layer chromatography
ICH	International Conference for Harmonization
CACI	Click Analytical Chemistry Index
RSD	Relative Standard Deviation
AGREE	Analytical GREEnness metric
BAGI	Blue Applicability Grade Index
UV	Ultraviolet
