# Supplementary material for: Stability-Indicating Spectrophotometric and TLC Densitometric Validated Methods for Simultaneous Assay of Salicylamide and Ascorbic Acid in the Presence of Salicylic Acid: Greenness Assessment and Practical Applicability
Source: Pharmaceuticals (Basel). 2026 Jun 24;19(7):980. doi: 10.3390/ph19070980 (PMC13414555; doi:10.3390/ph19070980)
Supplement: Supplementary file 1 [file pharmaceuticals-19-00980-s001.zip › pharmaceuticals-4361891-supplementary.pdf]

Stability-Indicating Spectrophotometric and TLC-densitometric Validated Methods for Simultaneous Assay of Salicylamide and Ascorbic Acid in the Presence of Salicylic Acid: Greenness Assessment and Practical Applicability

Omkulthom Al kamaly [1], Saja A. Althobaiti [2*], Maimana A. Magdy [3], Nourudin W. Ali [3], Hala E. Zaazaa [4], Mohamed Abdelkawy [4], Mohammed Gamal [3,*] and Maha M. Abdelrahman [3]

[1] Department of Pharmaceutical Sciences, College of Pharmacy, Princess Nourah bint Abdulrahman University, P.O. Box 84428, Riyadh 11671, Saudi Arabia
[2] Department of Chemistry, College of Science and Humanities in Al-Kharj, Prince Sattam Bin Abdulaziz University, Al-Kharj 11942, Saudi Arabia
[3] Pharmaceutical Analytical Chemistry Department, Faculty of Pharmacy, Beni-Suef University, Alshaheed Shehata Ahmad Hegazy St., Beni-Suef 62514, Egypt
[4] Pharmaceutical Analytical Chemistry Department, Faculty of Pharmacy, Cairo University, Kasr-El-Aini, Cairo 11562, Egypt
* Correspondence: s.althobaiti@psau.edu.sa (S.A) ; mgamalm3000@yahoo.com (M.G.)

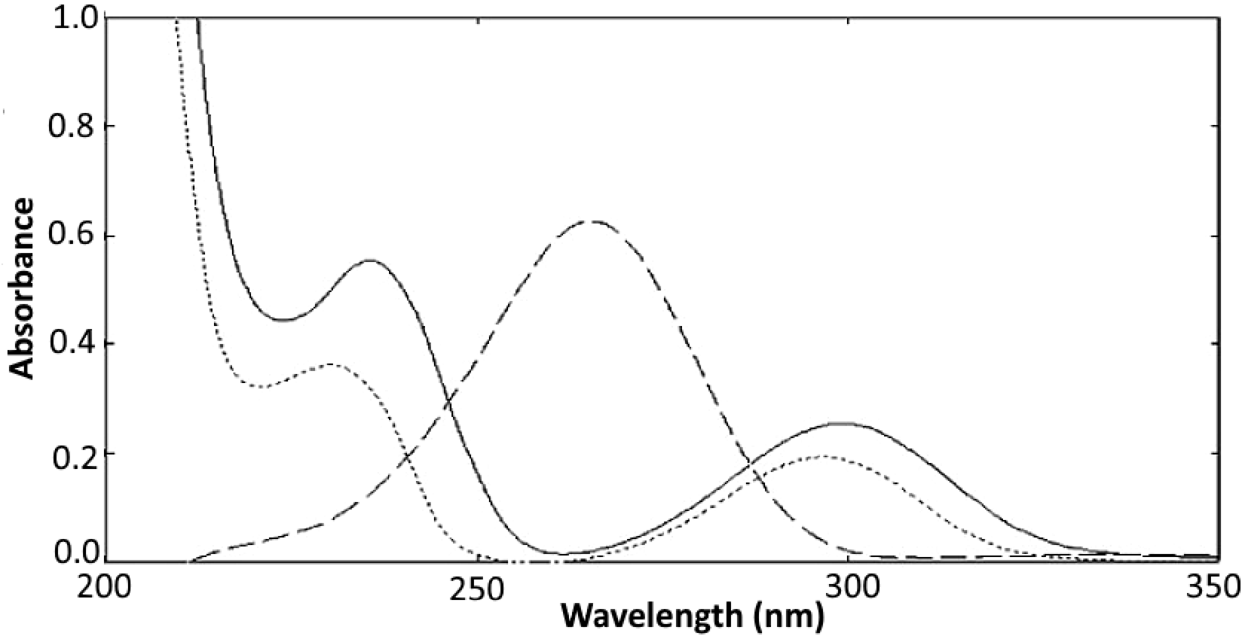

Supplementary Figure S1: Zero order absorption spectra of 10 µg ml^{-1} of each of Salicylamide (_), Ascorbic acid (----) ,and salicylic acid (….) using double distilled water as a solvent.

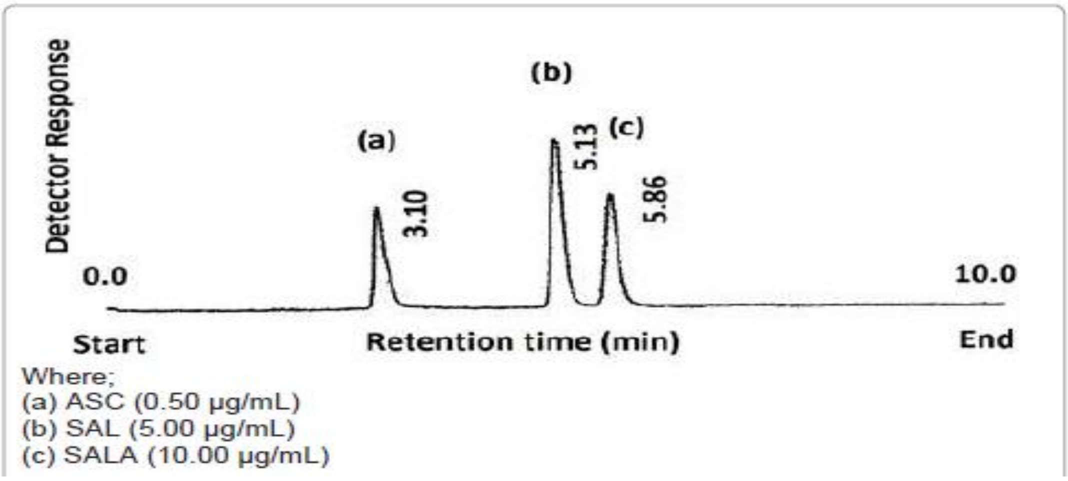

Supplementary Figure S2: A typical chromatogram for the lab-prepared mixture Ascorbic acid (A), Salicylamide (B), and salicylic acid (C) using C8 and chromatographic conditions reported by El-Din et.al. [35].

Supplementary Table S1: Experimental results of robustness for the assay of SAD, ASC, and SAL by the new TLC-densitometric technique. Robustness testing was performed for all mobile phase components except formic acid, which was treated as a critical item due to its high sensitivity; even minor volumetric changes substantially affected analyte responses and peak resolution.

**Parameters**	**TLC densitometric method (%RSD)**		
	**SAD (%RSD)**	**ASC (%RSD)**	**SAL (%RSD)**
**Chloroform (5ml ±0.1)**	0.521	0.731	0.513
**Hexane (3 mL ± 0.1)**	0.812	0.815	0.692
**Methanol (2 mL ± 0.1)**	0.941	0.915	0.632
**Acetone (1 mL ± 0.1)**	1.025	0.942	0.851
